# A tale of two controversies: situated AI frictions and the generative AI tsunami

**DOI:** 10.1007/s00146-026-03141-3

**Published:** 2026-08-04

**Authors:** Chiara Poletti, Noortje Marres, Donato Ricci, Christian Katzenbach, Lena Marie Henkes, Guillaume Dandurand

**Affiliations:** 1 Swansea University, Swansea, United Kingdom; 2 Centre for Interdisciplinary Methodologies (CIM), Warwick University, Coventry, United Kingdom; 3 Médialab Sciences Po, Paris, France; 4 Centre for Media, Communication and Information Research (ZeMKI), University of Bremen, Bremen, Germany; 5 Alexander von Humboldt Institut für Internet und Gesellschaft (HIIG), Berlin, Germany; 6 Institut national de la recherche scientifique, Montréal, Canada

**Keywords:** AI controversies, Generative AI, AI frictions, Public problematisation, Elicitation methods, Science and technology studies

## Abstract

What makes AI controversial? This article reports on an international expert consultation that we conducted between 2021 and 2023 to analyse recent controversies about AI in four countries: the United Kingdom, France, Germany, and Canada. Adopting a standpoint approach, this online consultation was designed to activate the collective intelligence of AI & Society expert communities to identify the most important but possibly overlooked controversies about AI during a 10 year period, 2012–2022. Based on our analysis of the consultation responses, we discern two contrasting discursive dynamics of problematisation in relation to AI during the relevant period: one revolving around the demonstration of “AI frictions”, the public staging of situated problems arising from the deployment of AI-based applications such as recommendation algorithms and image recognition in specific professional contexts and everyday environments. The other is associated with generalised and abstract forms of controversiality regarding generative AI technologies (GAIT), such as ChatGPT. Through a relational analysis of ‘topic-friction’ couplings latent in the consultation responses, we go on to distinguish two different types of ‘associational profiles’ (Marres 2015) for AI controversies: while situated AI frictions are defined by sustained connections with specific topics, GAIT controversies were marked by broad and thin connections across a broad range of topics. In capturing the decline and ascent of these AI controversy types, our consultation enables us to characterise a watershed moment in public debates about AI as a shift from deep to wide controversies. We conclude with the reminder that deep AI frictions have distinctive potential for democratic governance, because they tend to implicate organised communities and stakeholders with specific, demonstrable stakes in technology deployment. Wide GAIT controversies, by contrast, distribute concern across many domains in a diffuse manner, implicating general publics rather than affected communities, which creates special challenges for democratic accountability.

## Introduction

When OpenAI released its now iconic chatbot in November 2022, it marked a turning point in the public life of Artificial Intelligence (AI). However, in what way? Since its inception, AI has attracted cycles of attention, investments, optimism and disillusionment (e.g., “summers and winters”; Cardon et al. 2018; Roberge and Castelle 2020). Large Language Models (LLMs)—including earlier versions of the Generative Pre-trained Transformers (GPT) developed by OpenAI—had been the subject of intense interest among AI experts and the tech community for years before ChatGPT’s release. Furthermore, public attention to AI had moved in distinct waves well before then (Brennen et al. 2018; Dandurand et al. 2022; Marres et al. 2025). Yet, it was not until ChatGPT’s public launch in late 2022 that Generative AI Technologies (GAITs) reached mass uptake at a speed and scale that earlier developments had not achieved (Gordon 2023). However, GAIT did not only expand the use of AI from defined expert and professional spheres into manifold aspects of everyday life; it also changed the ways in which computational technologies are debated, problematised and publicised across society, becoming at the same time object and infrastructure of public discourse and policy^1^ (Ananny 2024; Crépel et al. 2025; König 2025; Marres et al. 2024; Suchman 2023).

But what precisely changed in the public politics of AI during this period, and how did this affect the engagement of publics, stakeholders and experts with technological politics and innovation governance? To address this question, we discuss a series of expert consultations about AI controversies conducted within the ORA project: *Shaping 21st Century AI,* between 2021 and 2023 in Canada, France, Germany, and the UK; a time frame that, as our analysis will show, sits at what appears in retrospect as a critical juncture in the public life of AI. The UK consultation (Autumn 2021) captured pre Chat-GPT AI critical expert discourse, while the consultations in France, Germany, and Canada (2023) captured the views of expert communities in the immediate wake of ChatGPT’s release, when the terms of controversy began to reflect generalising, promotional framings of GAIT. Our consultations, then, enabled us to analyse the events, processes and logics through which AI became the subject of public controversy in the four different countries, and how these dynamics shifted as GAIT became the defining phenomenon at the interface of AI and society from late 2022 onwards.

Our consultations implemented an explicitly contextual methodology. They were designed to ground our inquiry in situated expert knowledge rather than in the hyperbolic discourse that dominated media reporting on AI (Dandurand et al. 2022). At that time, this meant turning our attention to experts, policy-makers and activists who were actively involved in professional, public and critical debates about the topic of “AI and Society”, an area of expertise that is distinct from both AI scientists and from the far more expansive user community with proficiency in GenAI that would emerge later. In each of the four countries, we identified epistemic communities involved in the articulation of AI & Society issues, inviting experts in the social science, scholarship, policy, business, activism, and art of AI to tell us what they deemed controversial about it *from their perspective*. In our analysis of consultation responses, we examined both the themes and the types of controversy identified: did experts foreground legal controversies about accountability, for example, or incidents involving specific technologies, or existential issues raised by machine intelligence?

Our analysis enabled us to identify two different *types* of AI controversies across the different national contexts: 1) *deep* AI *frictions* (Marres et al. 2024; Shaffer Shane 2023; see also Kaun and Manniste 2025), which are inherently situated and context-specific and can act as catalyst for organised civic engagement and democratic governance and 2) *wide* AI controversies, prominently associated with GAIT technologies, whose mode of problematisation is abstract, generalised and de-localised, which implicate publics in a far more diffuse manner. The presence of these starkly different relational profiles of controversial AI in our controversy analysis, we will propose, indicate the emergence of GAITs acted as a *recomposer* of the controversy landscape (Lury 2020), ultimately altering the prevalent logics of the public problematisation of AI. We argue this recomposition of the controversy topology of AI has implications for the public politics of AI: while situated frictions implicate specific stakeholder communities, wide GAIT controversies are less likely to mobilise organised constituencies of AI in a similar manner, posing challenges for democratic governance.

The article is structured as follows. We begin in Sect. 2 by situating our expert consultation within the context of shifting discourses about AI as a public problem during the 2010s and early 2020s and the failure of AI controversiality to enable meaningful public contestation. We then present our contextual methodology (Sect. 3), grounded in standpoint approaches to the social study of science and technology (Harding 2009; Smith 1987), and outline our research design across four national contexts. Next, we present (Sect. 4) our data analysis, tracing connections and gaps across these contexts of AI controversy. Finally, in Sect. 5, we reflect on the implications of these patterns for understanding for the public problematisation of AI, and the challenges the rise to prominence of GAITs poses for the democratic governance of technology.

## Changing dynamics of public discourse about techno-science: what makes AI controversial?

Since the mid-2010s, advances in machine learning, neural networks, and large language models have sparked intense debate among scientists and experts (Cardon et al. 2018; Roberge and Castelle 2020). These controversies were accompanied by sensationalist media and public policy narratives, presenting AI as a historical turning point and driver of beneficial change for both human life and business (Brennen et al. 2018; Dandurand et al. 2023; Hansen 2022; Munk et al. 2024). National AI strategies and public policy institutes entrenched these promotional framings, positioning AI as inevitable and necessary (Bareis and Katzenbach 2021; Dandurand et al. 2022; Roberge et al. 2020; Paltieli 2022). These discourses privileged ‘strong AI narratives’ that project generalised and future-oriented imaginaries (Bory et al. 2024) while downplaying their potential to deepen structural inequalities (Munk et al. 2024; Powles 2018; Stilgoe 2018).

Over the past decade, AI has become a major topic of debate and controversy across academia, industry, public policy and the media in the countries under study. This trajectory was not linear. Following the more long-standing dynamics of AI summers and winters, in this phase, two major waves of public controversy surrounding AI have powerfully re-established what Lucy Suchman (2024) refers to as “uncontroversial thingness” of AI, now being considered an inevitable and defining characteristic of contemporary societies (Bareis and Katzenbach 2021). However, the initial wave of attention briefly receded during 2020, as the pandemic temporarily displaced AI from media agendas (Zeng et al 2026). From late 2022, however, the release of ChatGPT triggered a second and vastly larger wave of what has been once more characterised as a “tsunami” of promotional AI publicity (Marres et al. 2025; Roberge and Castelle 2020), embedding GAIT in everyday life, politics and culture at a speed and scale the earlier controversies had not achieved.

These dynamics contrast sharply with landmark cases of public controversy about science and technology of the preceding decades, such as climate change and genetically modified foods (Marres et al 2025), where STS research framed controversy across research, politics policy and activism as a force for the democratisation of science and technology, enabling the involvement of affected communities in evaluating their consequences for society (Callon et al. 2011). In this perspective, public controversy about science and technology can reveal the entanglement of social, political and technical relations, create pathways for the collective exploration and democratic settlement of public concerns (Latour 2004).

The trends highlighted in recent AI discourse, however, point in a very different direction: while public controversies about AI proliferated during the last 10 years, it is far from obvious that they resulted in the redistribution of agency from science and industry to policy, citizens and civil society actors. Rather, as AI scientists came to dominate media and government debates (Bareis and Katzenbach 2021; Natale and Ballatore 2017), public discourse consolidated the epistemic authority of science and industry and the agenda-setting power of tech companies. Discourses on AI ethics and responsibility thematised some concerns of privacy, transparency, fairness and discrimination, but in many respects, failed to create opportunities for the translation of civic engagement into democratic policy frameworks (Hirsch-Kreinsen 2024), remained confined within narrow technological frameworks (Panarese et al. 2025) or did not address the underlying concentration of power among dominant corporations (Bode and Goodlad 2023; Kalluri 2020).

It was against this background that we undertook a consultation with *AI & Society* experts from 2021 onwards to answer the question “what makes AI controversial?”. We were curious about experts’ views and assessments of the most important AI controversies of the previous decade, and which features of recent AI controversies had retained the capacity to support the public problematisation of AI science and innovation and their role in society.

## Methodology: a standpoint approach to controversy elicitation

As public discourse about AI seemed to consolidate power asymmetries, we adopted a design approach for our consultation that was well-suited to this type of situation: standpoint methodology. Developed by feminist scholars of science and technology, such as Dorothy Smith (1987) and Sandra Harding (2007, 2009), this approach foregrounds lived experiences, specific contexts and epistemic communities involved in the development, deployment and practice of science and technology. Following this commitment, our aim was to ground our controversy analysis in specific, situated practices, communities and settings, attending to local and context-specific framings of AI and its controversiality to recover its generative capacity (Gourlet et al. 2024; Jaton and Sormani 2023; Marres and Sormani 2023). To operationalise this commitment, we adopted a methodology of “controversy elicitation” (Marres et al. 2024): we identified situated communities of experts already involved with AI & Society issues to elicit their contextual understandings of what makes AI controversial from the ground up. Differently put, we used a call-and-response methodology (Puwar and Sharma 2012) to look beyond the promotional discourses circulating in publicity media. This also means that we adopted a deliberatively selective approach, seeking to identify a particular type of AI controversies, those with the capacity to problematise “AI” on epistemic, normative, or empirical grounds. The objective here was less to offer a comprehensive overview of the AI controversy landscape than to detect and render explicit increasingly rare, distributed processes of problematisation of science and technology in society (Marres and de Rijcke 2020; Ricci 2019). Accordingly, our guiding question of “what makes AI controversial?” inherently implied another: “controversial for whom?”.

## Research design: consulting with extended expert communities across national contexts

Our consultation design relies on a deliberately broad definition of expertise. In turning to AI & Society experts to identify important but overlooked controversies about AI, our aim was precisely *not* to restrict controversy to certified knowledge, but to engage less dominant voices. For this reason, we adopted an inclusive definition of expertise, one that includes experts by experience, according to the definition Funtowicz and Ravetz (1991: 170) provide of the “extended peer community,” as “all those with a stake in an issue who are committed to genuine debate.” Our recruitment strategy focused on a diverse set of AI & Society events (more on which below), which, at the time of the consultations, constituted some of the primary sites for exchange and communication among actors engaged with AI-based technologies as a force for societal change. This allowed us to include a plurality of types of experts, from more traditional AI & Society researchers and policy experts to those engaging with AI from community and artistic standpoints.

Operationalising controversy analysis through expert consultation also enables its implementation in a cross-national research design. We asked: how was the controversiality of AI articulated differently across epistemic communities in the different national contexts? Our comparison, though, is not structured in terms of what Ulrich Beck terms “methodological nationalism” (Beck 2007). In adopting a standpoint approach, we seek to resist the tendency to homogenise expert communities across national contexts and affirm differences and incommensurabilities between them. We aimed for a type of discourse analysis that respects the distinct and context-dependent nature of each epistemic community’s standpoint and affirms the complex variation in the settings of AI and society controversy across national boundaries. However, as it turned out, alongside the differences, our analysis revealed common patterns across the national contexts.

**Fig. 1:**
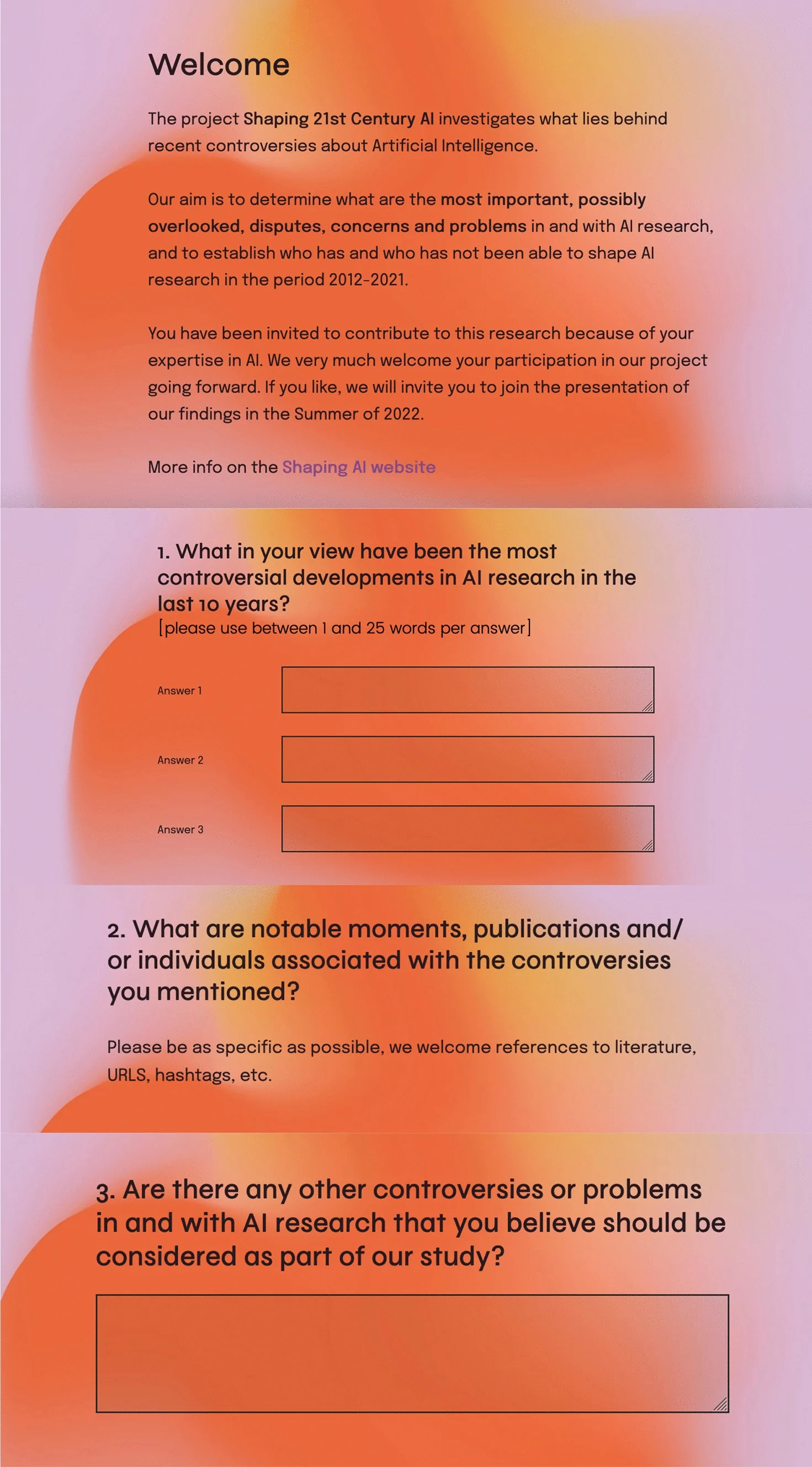
The interactive digital platform (Qualtrics) used during the consultation process

We chose online consultations for their ability to capture culturally and contextually specific concerns across countries (O’Brien et al. 2016), as well as for their flexibility, which combines elements of questionnaires, interviews, focus groups, and workshops (Dendler et al. 2023; Mayo et al. 2021). We adopted the questionnaire as the preferred initial format, as it allowed us to configure controversy elicitation as a responsive process. We were aware that consultations face challenges in this regard, as their design may constrain participants’ ability to introduce new issues or potentially limit spontaneous engagement (Delgado et al. 2023; Hintz et al. 2022; Sloane et al. 2022). To avoid imposing predefined agendas and allowing participants to define key issues in their own terms (Delgado et al. 2011; Marres 2007), we structured our consultation around three open-ended questions (see Fig. 1):

A) What in your view have been the most controversial developments in AI research in the last 10 years?
B) What are notable moments, publications and/or individuals associated with the controversies you mentioned?^2^
C) Are there any other controversies or problems in and with AI research that you believe should be considered as part of this study?

Regarding recruitment of experts, we conducted two separate consultations in each country, one with AI & Society experts and another with computational scientists, because we realised these constitute largely separate epistemic communities. In this article, we only report on the findings of the AI & Society consultation.^3^ For both groups, the research teams in the respective countries identified relevant venues where expert discussions took place between 2019 and 2023. These were events, such as conferences, workshops and public forums. Treating such events as standpoints, we selected experts from registration lists and associated publications (see Table 1 for more details).^4^ Some teams, such as France, additionally relied on social media debates to identify relevant experts, while for others, such as Canada, the process was complemented by recruiting participants with relevant institutional affiliations (e.g., MILA, IVADO, AMII, and OBVIA). In this way, the delineation of our expert communities was at once substantive (i.e. identifying experts in AI & society), country-specific (grounded in UK, France, Canada, Germany) and medium-based (conference lists, institutional affiliations, social media debates). Participants were selected on the basis of:

- Engagement with AI & Society research or staying informed about AI advancements as critical to their professional role; or
- Involvement in knowledge production and intervention, including work aimed at shaping or contesting public understandings of AI in society; or
- Active participation in AI knowledge spaces, particularly in roles aimed at influencing public knowledge on AI & Society issues.

**Table 1:** Overview of national consultation rounds: context, recruitment, and participation

Team	Consultation period	Recruitment and composition of expert community	Experts invited	Respondents	Response rate (%)
UK	Autumn 2021 (Nov 2021)	Recruitment focused academic, business-oriented, and activist events, (e.g., CogX, the Turing Lectures, Mozilla Festival, and ArtAI Festival)	250	53	21.2
France	Spring 2023 (Feb 2023)	Twitter-based recruitment strategy with contributors focused on public sector applications, accountability, and socio-technical issues	69	19	27.5
Germany	Spring 2023 (Feb 2023)	Event-based recruitment with additional outreach; primarily academics, NGO representatives, and stakeholders with expertise in governance, automation, and digital rights	124	35	28.2
Canada	Summer 2023 (Jul 2023)	Combined event-based and institution-linked strategies; diverse group of public sector professionals, civil society actors, and policy-engaged researchers	136	20	14.7

The expert communities we consulted through these events spanned policy and regulatory circles, journalism, and STS scholarship as well as artists, activists and practitioners whose engagement with AI operated outside academic, expert and policy frameworks. In the UK, events such as ArtAI Festival and the UAL Technology and Power seminar explicitly reached beyond academia. However, even if not restricted to specific institutions, these events did not yet reflect the kind of diffuse, everyday AI expertise that now circulates widely across different practitioner communities and publics. If this research were conducted today, we would operationalise the “extended expert community” in a very different manner, as concern with AI as a force of social change has become generalised, and many citizens can be expected to have informed views on the matter. We return to the implications of this sampling choice in Sect. 6.

### Relational analysis: topic-friction couplings

**Fig. 2:**
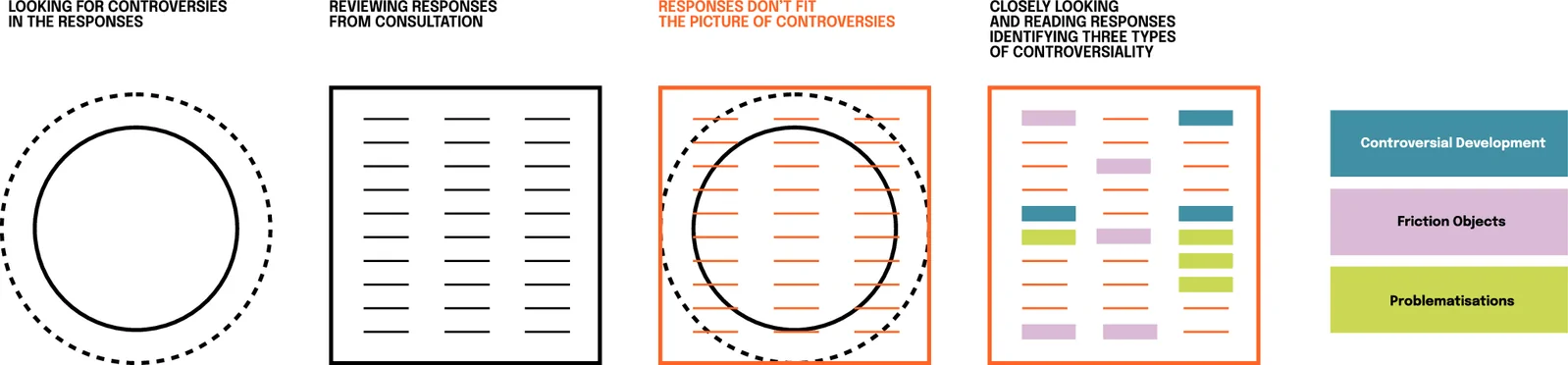
Iterative categorisation of the consultation results

Our iterative categorisation of the consultation responses (see Fig. 2) raised questions about the defining features of AI controversies as compared to other forms of publicity relating to AI, such as debate, scandal, and hype.

In the sociology of science, controversy is typically defined as staged disagreement about specific points of contention between experts (Collins and Pinch 2014; Latour 1987; Rogers and Marres 2000) but we found relatively few instances of these clean-cut instances among the responses. Rather, our consultation surfaced several different types of expressions of AI’s controversiality: broad controversy *topics* relevant to AI, specific controversial incidents (*frictions*) involving AI, and more open-ended problem statements (*problematisations*) (Table 2).

**Table 2:** Different types of AI’s controversiality

Category	Definition	Example
Topics	Thematic areas of prominent debate and dispute in relation to AI	Algorithmic decision-making; machine bias
Frictions	Specific incidents, sites, applications or systems where AI causes demonstrable disruption or tangible contestation	Dismissal of Timnit Gebru; wrongful arrests from facial recognition
Problematisations	Statements articulating what the problem with AI is; societal tensions and risks	“AI ethics’ failure to confront corporate power”; “erasure of disabled people from training data”

**Fig. 3:**
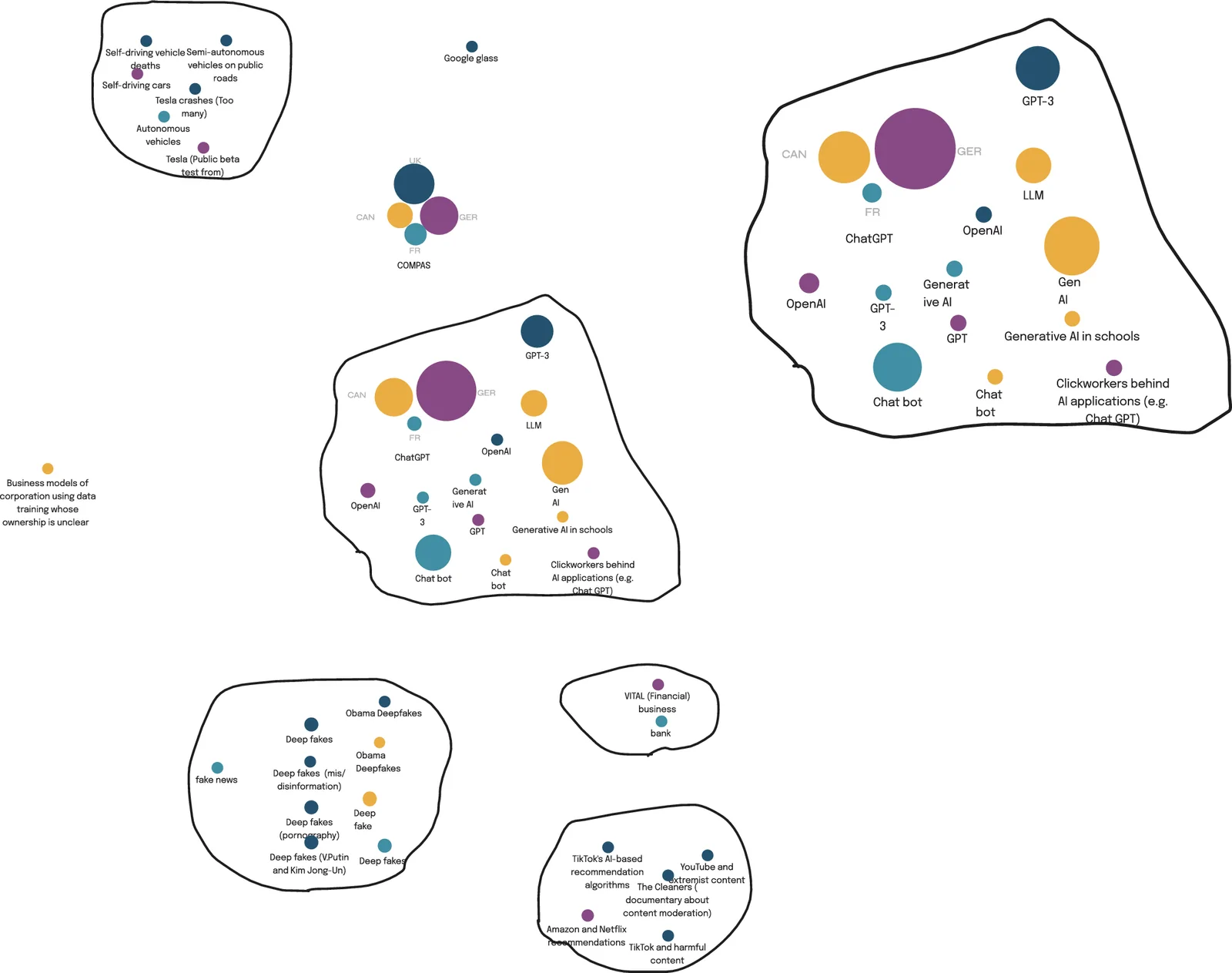
Collaborative clustering of AI frictions. This figure presents a screenshot of the online Figma environment in which the four national research teams collaborated to code friction-topic couplings, supported by a video-meeting. The visual clustering shows a portion of the map generated through collaborative coding and clustering, illustrating the distribution of AI frictions and associated topics across all national contexts; the full map contained all 18 friction clusters and 12 topic clusters identified through our analysis. The top right shows the internal structure of the LLMs cluster. Circle size indicates frequency of mention, and colours denote national origin of the codes

We first conducted a frequency analysis of the occurrence of these different types (reported in Marres et al. 2024) and explored their relations in a three-stage process (Table 3): did respondents mention *frictions* in relation to some *topics* rather than others? Which *problematisations* were mentioned in relation to which *frictions* and which *topics*? This relational analysis is inspired by the scientometric method of analysing *heterogeneous couplings*, a form of data analysis grounded in co-word analysis and bibliometric clustering (Waltman et al 2010), which maps “the co-occurrence of science and non-science objects, actors, and interactions” (Costas et al. 2021, p.4). This allowed us to analyse the controversiality of AI as involving the association or ‘drawing together’ of diverse entities, deemed heterogeneous insofar as they may derive from different spheres (e.g., science, government policy, and civil society). They are not controversial by default; rather, they may contribute to the creation of controversy through their combination. By mapping such *topic-friction* couplings, this study draws on topological approaches (Lury 2020; Marres 2012; Stirling 2019): our aim was to capture the shifting relational composition of AI “problem spaces”, as they bring topics, incidents and problems into relation, reflecting historical contingencies, national policy agendas and global debates Fig. 3.

**Table 3:** Three stage analysis of heterogeneous couplings

Stage	Process
1. National coding	Each national team coded AI frictions, topics, and problematisations from consultation responses
2. Cross-national clustering	Comparative clustering via iterative collaborative discussion and visual mapping (Figma) to identify transnational patterns (see Fig. 3). We established 18 clusters of AI *frictions* and 12 clusters of *topics*
3. Coupling analysis	Co-occurrence analysis of friction-topic pairs, combining quantitative patterns with qualitative interpretation, media analysis, and expert interviews

## Findings

This section presents the main findings from our relational analysis of the four national consultations. While our analytical process began with country-specific coding (as described in Table 3) to facilitate the interpretation of cross-national differences, we start our discussion with an overview of how AI *frictions* and *topics* appear across all four countries (Sect. 4.1). We then examine their couplings in this aggregate form (Sect. 4.2), before turning to national variations (Sect. 4.3). We review these variations considering each consultation’s situated standpoint in time as much as in place.^5^

### Overall distribution: the prominence of GAIT, surveillance and ethical concerns

**Fig. 4:**
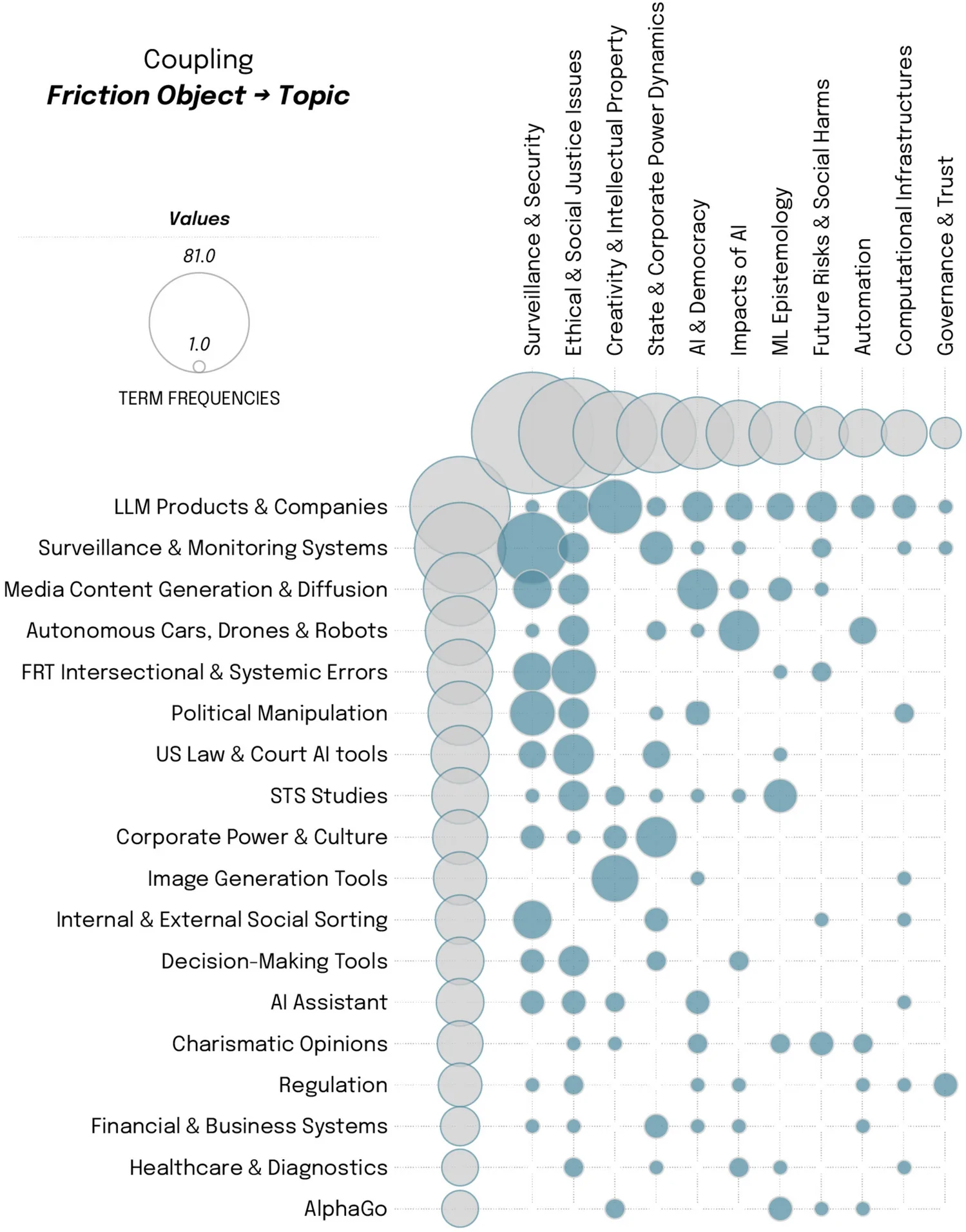
Bubble matrix of frictions. Topics couplings (aggregate view across all countries). This figure represents the frequency of co-occurrences (i.e. couplings) between AI frictions (rows) and controversy topics (columns) across the four national consultations. Bubbles on x and y axis represent the total number of references offering a measure of their overall prominence

The cross-country frequency of AI *frictions* (*y*-axis in Fig. 4) reveals an uneven distribution. A small number of highly visible systems, particularly general-purpose GAIT and surveillance technologies, account for most expert mentions, while domain-specific applications receive comparatively fewer mentions. “LLMs Products & Companies” tops the list and is the most frequent category by a significant margin. This includes general-purpose tools, such as Codex, ChatGPT, and GPT-3, which have come to exemplify the latest wave of generative AI. Following closely are “Surveillance & Monitoring Systems”, encompassing tools based on facial recognition and biometric tracking. Two further GAIT-related categories, “Media Content Generation & Diffusion” (29 mentions) and “Image Generation Tools” (15), also rank among the most recurrent. Other frequently mentioned categories include “Autonomous Cars, Drones & Robots” (26), “Facial Recognition Technologies (FRT) Intersectional & Systemic Errors” (23), and “Political Manipulation” (22), referring to cases, such as Cambridge Analytica. While more domain-specific frictions, varying from “AI Assistants”, such as Siri, Alexa, to sector-specific AI systems in healthcare, law, insurance and financial services, are less frequent.

The distribution of *topics* (*x*-axis of Fig. 4) reflects the shared thematic priorities and interpretive orientations of the expert communities consulted. The top four topics account for nearly two-thirds of all topic references. The most prominent is “Surveillance & Security” (81 mentions), including concerns about biometric identification, predictive policing (e.g., COMPAS and Palantir), social profiling, border control systems (e.g., iBorderControl), and other forms of AI-enabled tracking. This is followed by “Ethical & Social Justice Issues” (66), with debates over algorithmic bias (e.g., Gender Shades study, representational harms in image tagging), privacy violations, discrimination in hiring algorithms, and fairness. In third place is “Creativity & Intellectual Property” (38), focused on authorship, copyright disputes (e.g., Midjourney in Colorado art contest, DALL·E), and the appropriation of training data in generative AI systems. The fourth most frequent topic, “State & Corporate Power Dynamics” (34), includes concerns about opacity, concentrated influence, and accountability gaps in both corporate-led AI development and public–private governance frameworks. By contrast, technically bounded topics such as “Automation” (12 mentions), “Computational Infrastructures” (11), and “ML Epistemology” (6) are much less frequent among the responses. The relatively low frequency of technical topics suggests that, for the experts consulted, the most pressing issues raised by AI are not internal to its technical architecture, but lie in its implementation, regulation, and societal implications.

### Topic-friction coupling analysis: deep vs. wide AI frictions and their topics

We now turn to the co-occurrence of topics and frictions.^6^ The bubble matrix in Fig. 4 serves as a starting point for tracing the couplings. The size and colour of each bubble capture the frequency and strength of each association, while their distribution across each row reveals how each friction object connects to a variety of topics. This dual representation allows us to identify different connectivity profiles, characterised by two attributes: spread, i.e., how many different topics a friction object links to, and strength, i.e., how frequent those links are. On this basis, we distinguish between wide and deep connectivity profiles.

At one end of the spectrum are what we term *wide* frictions, characterised by relatively weak links to a broad range of topics. These appear as rows with many bubbles of comparable size, indicating a relatively evenly distributed and generalised pattern of linkage across multiple topics. In our data, LLMs illustrate this profile: they are coupled with all twelve topics, with relatively stronger links to “Creativity & Intellectual Property”, “Ethical & Social Justice Issues”, “AI and Democracy” and “Future Risks & Social Harms.” This wide distribution reflects not only the general-purpose nature of these technologies, but also a higher level of abstraction in how respondents described them, rather than through references to specific incidents or deployments that mobilise interest groups.

At the other end of the spectrum are ‘deep’ AI *frictions*, defined by strong links to fewer topics*.* These appear as rows with one or two markedly larger bubbles alongside much smaller or absent ones, pointing to a concentrated topical anchoring. These AI frictions are more clearly tied to named deployments in specific institutional settings and documented incidents involving identifiable actors and sites: a specific hospital’s data transfer, a named police force’s facial recognition trial (e.g., King’s Cross, London; Südkreuz, Berlin), or a particular company’s discriminatory hiring algorithm. These cases are also those often associated with the formation of organised public coalitions and, in several instances, with regulatory or legal responses.

### Country-specific findings

We now examine how these couplings occur and vary across national contexts, reflecting on how institutional context, public discourse, and consultation timing shaped which topics and AI frictions were salient, as well as the groundedness of their problematisations.

**Fig. 5:**
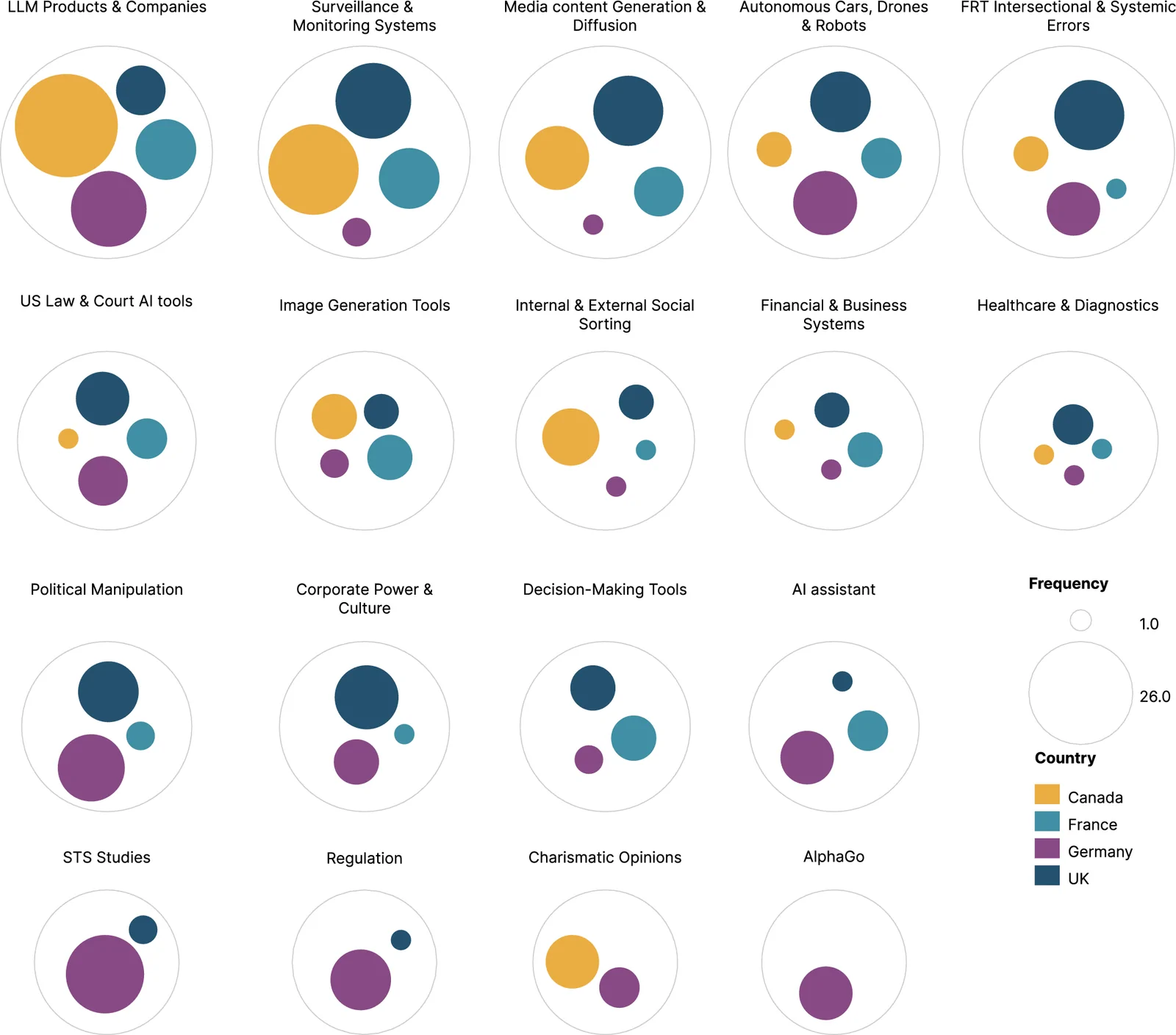
AI Frictions distribution across countries

Figure 5 shows how friction objects are distributed across countries, revealing both transnational and nationally embedded dynamics. Some frictions (e.g., LLMs, Surveillance and Monitoring Systems, Media Content Generation, FRT Intersectional Errors) appear across multiple contexts. Others are nationally specific: “Political Manipulation” appears only in Germany, France and the UK; “Charismatic Opinions” only in Canada and Germany. This uneven distribution points to the interplay between globally circulating AI imaginaries and the situated concerns that shape each national consultation.

To understand how specific frictions are defined within each national context, we analysed the coupling between friction objects and topics using TF-IDF weighting 7.

#### United Kingdom (Autumn 2021)

**Fig. 6:**
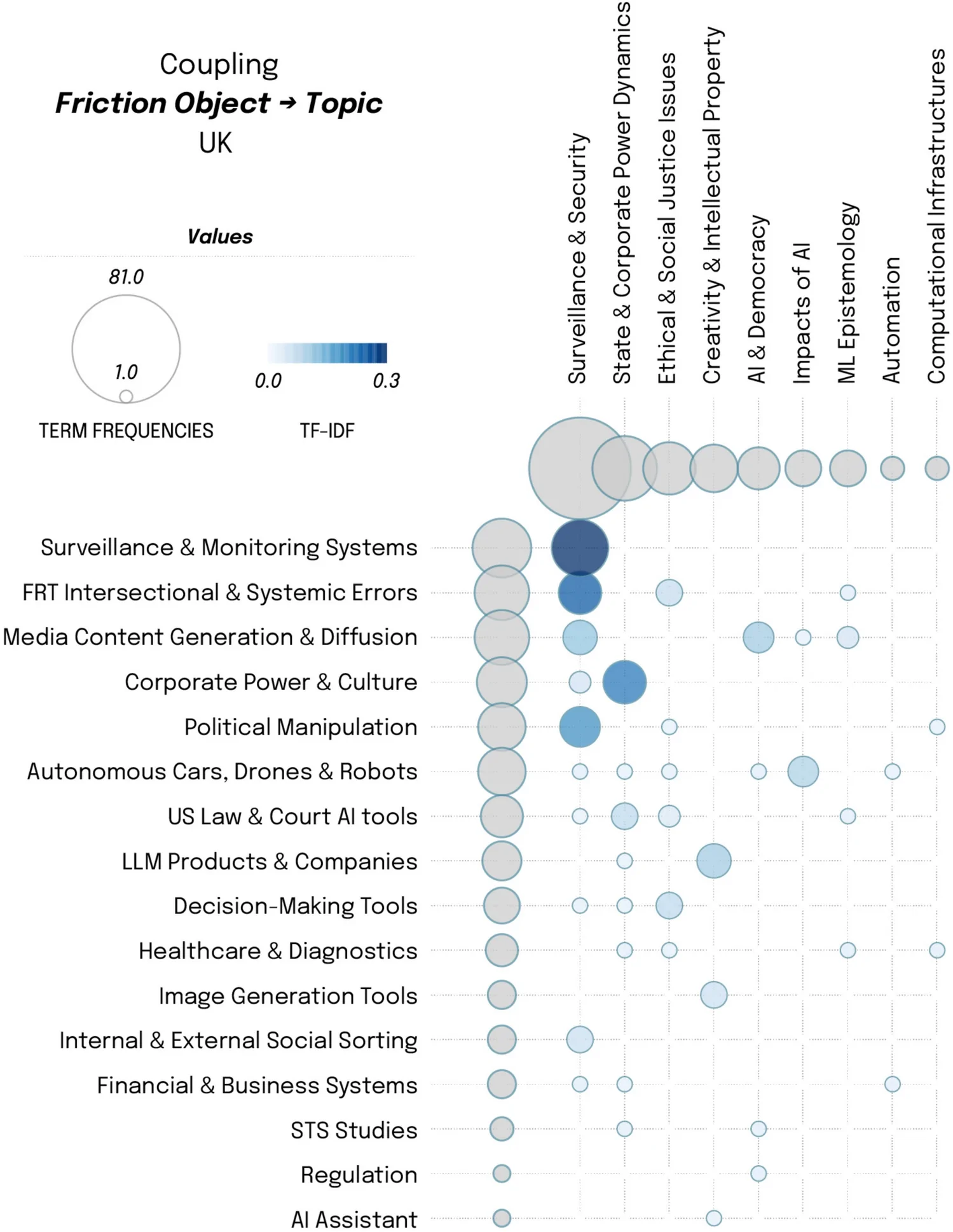
UK AI frictions—topic coupling frequencies. The figure shows the couplings ordered by relevance in the UK

The UK consultation was conducted in the Autumn of 2021, at a time when the media debate about AI in the UK was largely shaped by industry actors and a relatively narrow set of expert voices (Brennen et al. 2018). The public discussion had not yet reached the scale and intensity that would follow the large-scale adoption of ChatGPT (Roe and Perkins 2023). The moment was marked by a particularly active controversy around facial recognition technology: events such as the deployment of facial recognition by South Wales Police in 2018 and its use by the Metropolitan Police at King’s Cross station and the Notting Hill Carnival (Sabbagh 2019) had raised concerns among experts and civil society. Organisations such as Big Brother Watch and Liberty had been mounting sustained public challenges to the police’s use of the technology, and the Information Commissioner’s Office (ICO) had intervened in October 2021 (ICO 2021), providing an institutional basis for regulatory intervention. Consequently, four out of the five most contextually relevant couplings centre on surveillance, security and discrimination concerns (Fig. 6). This distribution reflects the governance potential of deep topic–friction couplings: by anchoring concern in a specific, contestable object, the controversy generated organised pressure which produced a concrete regulatory outcome.

A second cluster of deep frictions emerged around “Healthcare and Diagnostics”, where experts referred to cases such as DeepMind’s partnership with the Royal Free London NHS Foundation Trust, deemed unlawful by regulators in 2017 (Hern 2017), alongside the Google Nightingale controversy, and the Care.data project. These cases mobilised a coalition of privacy advocates, legal scholars, and healthcare professionals around questions of data ethics and public–private data infrastructure and institutional accountability (Powles and Hodson 2017). By contrast, “LLM Products and Companies”, a ‘wide’ AI friction in the overall overview from Fig. 4, in the UK showed only one strong link connecting it to “Creativity & Intellectual Property”, with only weaker ties to issues of power or governance through the reference to the Gebru case (Hao 2020), which at the time of the UK consultation, had not yet translated into broader public or policy debate around GAIT.

#### France (Spring 2023)

**Fig. 7:**
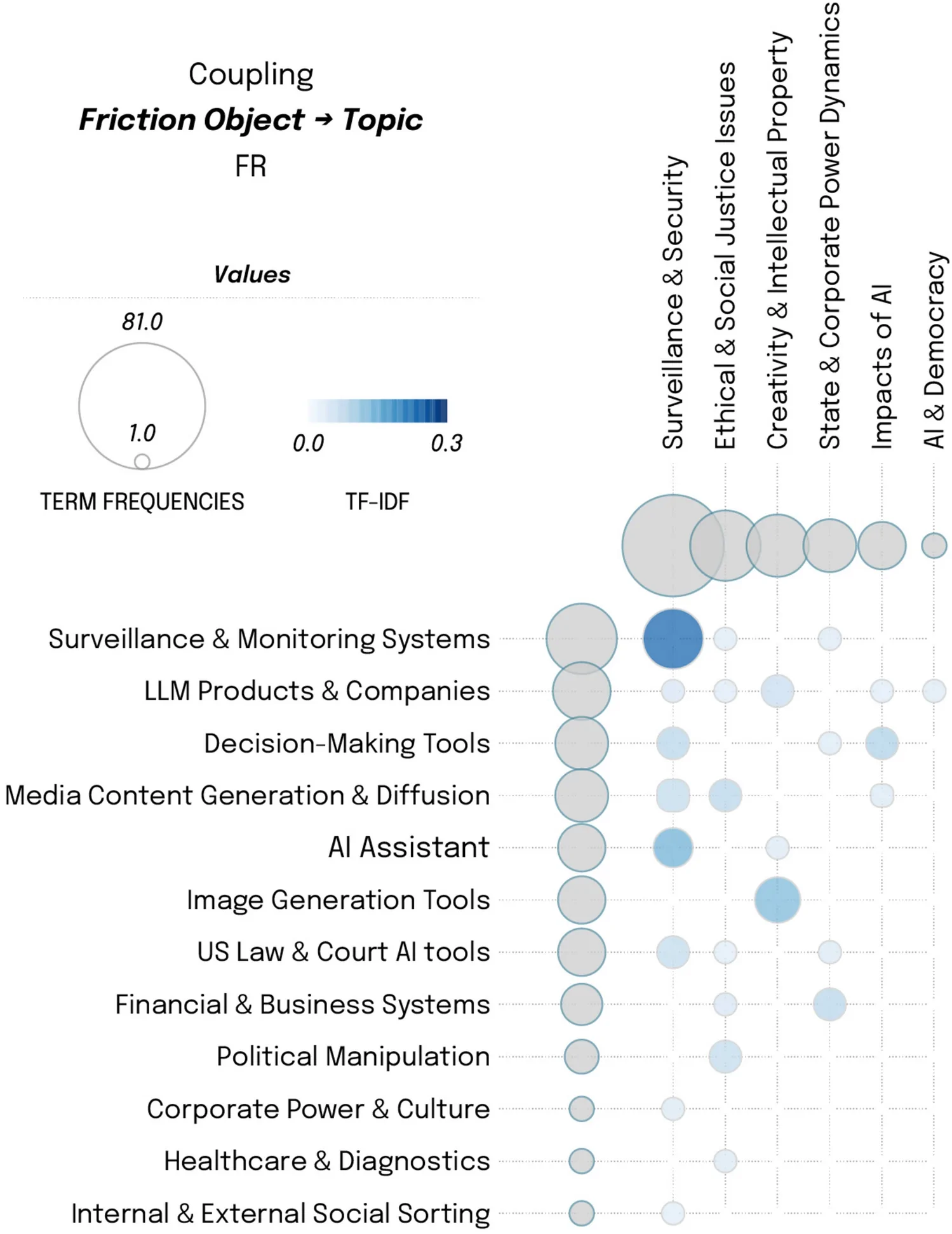
France AI frictions—topic coupling frequencies. The figure shows the couplings ordered by relevance in France

Conducted in Spring 2023, shortly after ChatGPT’s release, the French consultation captured a moment in which GAIT was gaining topical breadth but had not yet displaced the situated frictions that had been the prominent foci of AI controversy in France. Surveillance systems featured as a dominant issue (Fig. 7): the coupling “Surveillance and Monitoring Systems” + “Surveillance & Security” highlights public concern around AI deployment in urban public spaces. The legal prohibition of facial recognition systems in schools in Marseille and Nice in 2020 were particularly prominent cases, underscoring the nation’s cautious approach to biometric surveillance (Quadrature and du Net 2020).

A uniquely French concern emerged in the linkage of “Decision-Making Tools” with both “Surveillance & Security” and “Impacts of AI.” This coupling centred on the *Caisse Nationale des Allocations Familiales* (CNAF) welfare fraud detection algorithm, which assigned risk scores to beneficiaries and generated allegations of discrimination against marginalised groups, including individuals with disabilities and single mothers (Quadrature and du Net 2022). This deep friction culminated in formal legal contestation in 2024, when a coalition of 15 organizations, led by La Quadrature du Net and Amnesty International, filed a complaint with the Conseil d’État, France’s highest administrative court, challenging the algorithm’s legality and its compliance with data protection regulations (Amnesty International 2024).

By contrast, LLMs in France were coupled to issues of surveillance, profiling, disinformation, democratic disruption, algorithmic bias, and labour conditions. LLMs in France thus showed a wider topical profile than in the UK, but lacked the situated specificity that, in other cases, enabled organised contestation of specific deployments.

#### Germany (Spring 2023)

**Fig. 8:**
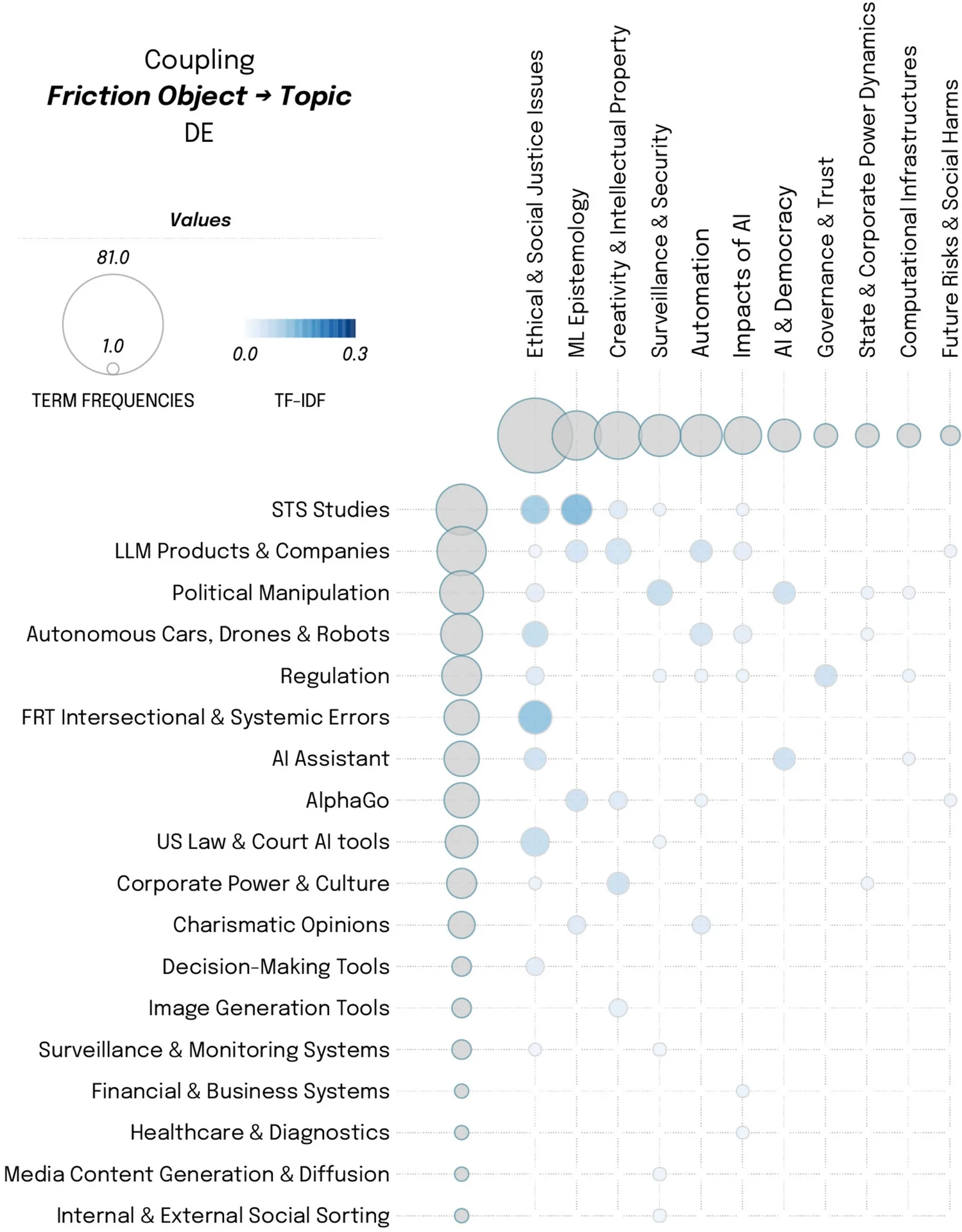
Germany AI frictions—topic coupling frequencies. The figure shows the couplings ordered by relevance in Germany

The German consultation, also conducted in Spring 2023, revealed a distinctive mode of AI’s controversiality shaped by academic and regulatory framings. The top-ranked coupling “STS Studies” + “ML Epistemology” reflects an expert community oriented toward critical theory and interpretive approaches to technology^8^ (Fig. 8). Two other top couplings connect “FRT Intersectional & Systemic Errors” and “STS Studies” with “Ethical & Social Justice Issues,” but unlike the UK or France, these concerns were expressed as conceptual critiques, rather than domestic incidents, reflecting Germany’s strong regulatory restrictions on real-world use of FRT. “US Law & Court AI Tools” + “Ethical & Social Justice Issues”, centred on the COMPAS recidivism algorithm, ranked highly despite these tools not being used domestically, suggesting German experts were drawing on international deployment examples to flag risks and governance issues. A further distinctive trait of the German findings is the ways in which topics linked with “Regulation (e.g., the AI Act or GDPR)” ranged from democracy and governance to surveillance, ethics, automation, and computational infrastructure, probably reflecting EU-level debates on the AI Act effects on national AI governance. Taken together, these patterns reveal a distinctive German trend, in which experts drew on foreign deep frictions as proxies to articulate governance concerns, while, domestically, pre-existing frameworks absorbed and structured critical concerns at the institutional level. Where the UK and French cases show public mobilisation around specific local deployments, the German case shows regulatory architecture mobilising governance discourses through institutional structures (Jones 2022; Löblich and Wendelin 2012).

LLMs appeared across six topics, from epistemology, future risk, labour, bias, and education but always with low relevance and without connection to specific incidents, confirming wide frictions do not elicit grounded contestation even when topically expansive.

#### Canada (Summer 2023)

**Fig. 9:**
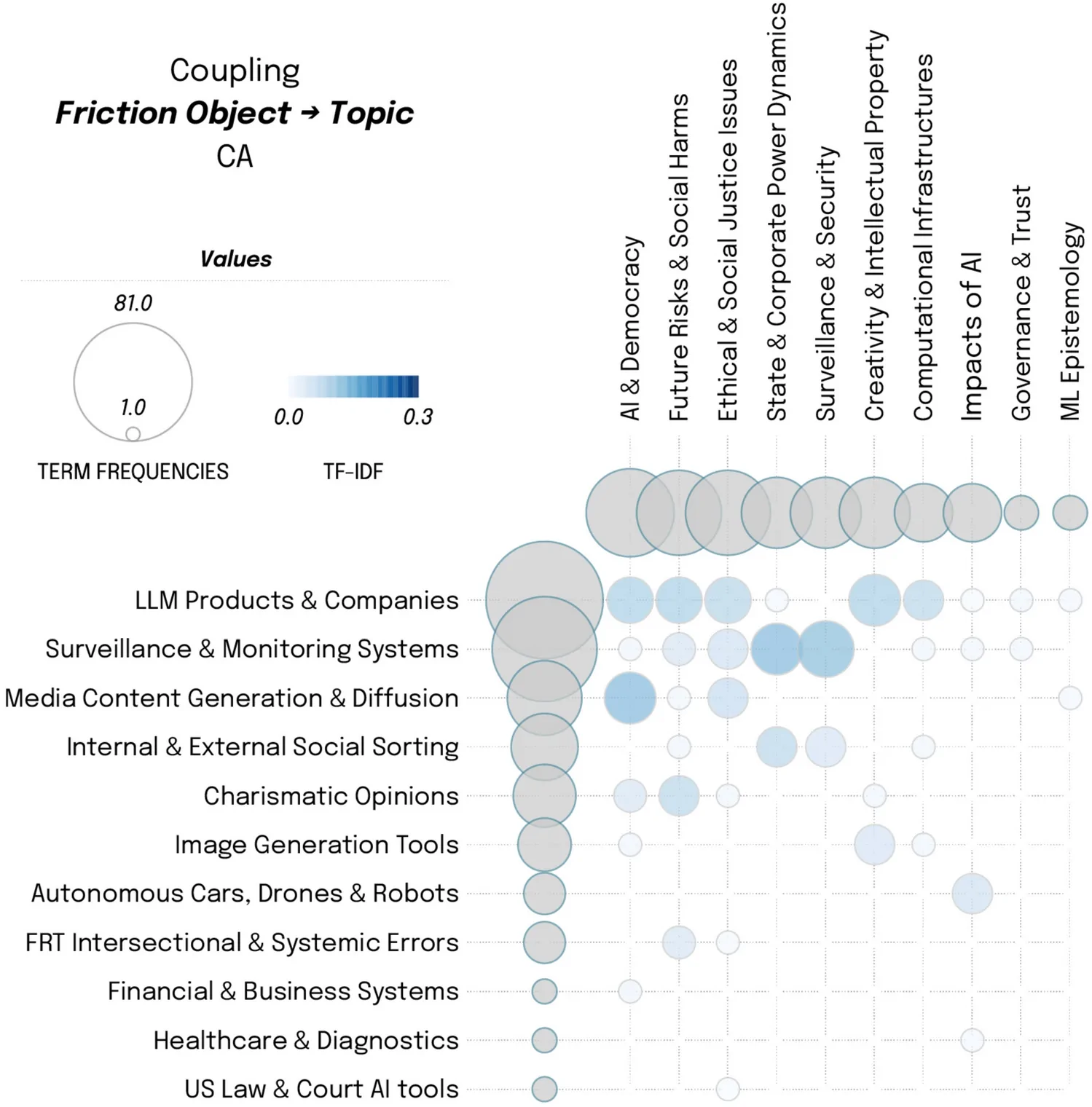
Canada AI frictions—topic coupling frequencies. The figure shows the couplings ordered by relevance in Canada

The consultation in Canada was conducted in Summer 2023 and captured a community more reorganised around GAIT, producing the widest controversy profiles overall: a broadly distributed landscape in which GAIT-related and surveillance-related couplings shared the top ranks without either achieving the topical anchoring visible in the earlier consultations (Fig. 9). The leading couplings “Surveillance and Monitoring Systems” + “State & Corporate Power Dynamics” and “Media Content Generation and Diffusion” + “AI & Democracy”, reflect concern over public–private AI infrastructure and information integrity, but in generalised rather than situated terms: Clearview AI and deepfakes feature as reference point but without the institutional specificity. More broadly, this overall controversy profile appears to be consistent with Canada’s position at the periphery of the American technological complex; a circumstance that would later translate into concerns about data sovereignty, for instance.

LLMs were an especially salient issue in Canada, in part due to the timing of the consultation in the post-ChatGPT period, but also in view of a specific framing on the systemic and existential stakes in the contestation of AI innovation. Compared to other countries, LLMs here appear thematically broader and more relevantly coupled across a significant number of high-ranking topic clusters. These span ten topic clusters ranging from democracy and future risks to corporate power and social life. The Canadian consultation responses can be taken to reflect the growing influence of Canadian AI scientists such as Yoshua Bengio and Geoffrey Hinton on public discourse. Their public interventions (e.g., the open letter on AI risks) shifted the discourse toward existential framings, surfacing in the coupling “Charismatic Opinions” + “Future Risks & Social Harms.” National spokespeople who advocated general, at times vague and non-binding concepts such as “responsible” AI here attracted significant public interest (Chartier-Edwards et al. 2024). What emerged was a form of concern distributed across the broadest possible range of topics and expressed through generalised framings rather than through contestation of specific systems.

The governance consequences of this profile are most visible in what the Canadian dataset does not contain. High-profile domestic cases such as Sidewalk Labs, Element AI, or the Pan-Canadian AI Strategy are largely absent. In a context where AI controversy had been systematically framed as a “cold controversy” by media and institutional actors (Dandurand et al. 2023), the Canadian case illustrates how GAIT framings appear associated with the marginalisation of the situated frictions through which democratic accountability of AI might otherwise become actionable.

## From deep to wide: shifts in the profiles of AI controversy objects after ChatGPT

Our findings reflect both the composition of the expert communities we consulted in each country and the timing of each consultation, but they also disclose a broader shift demarcating the period “before” and “after” the release of ChatGPT. This finding loosely maps on the distinction made by Bory et al. (2024) between “strong” and “weak” AI narratives: between narratives that assign generalised, often humanlike or superhuman, faculties to AI (strong) versus representations of narrow AI applications and their functions in concrete contexts in the present (weak). We propose, however, to conceptualise this difference differently: rather than strong versus weak, we frame it as the contrast between two types of controversiality that arise from wide versus deep connections among, on one hand, technologies or incidents, and, on the other hand, concerns, concepts, or ideals.

Across all four national contexts, some AI frictions, such as Facial Recognition technologies, functioned as paradigmatically *deep*: anchored to named deployments, specific legal disputes, identifiable institutional actors and identifiable affected communities. Their controversiality corresponded to a strong concentration on fewer topics. By contrast, GAITs, and LLMs in particular, displayed the inverse *wide* profile. LLM couplings expanded across the consultations, from a single topic in the UK (2021) to ten in Canada (2023), though this proliferation of topics was rarely matched by deep situational grounding. Across countries, LLMs problematisations remained predominantly abstract, circulating through generalised topic frames rather than specific incidents.

These differences can be made legible as a tale of two types of controversies: before ChatGPT, AI’s controversiality was expressed in distributed yet situated frictions; while after the most recent wave of AI hype, it involved the diffusion of abstract and generalised assertions. Our data thus capture a particular shift in the structure or topology (Lury 2020) of AI controversies following ChatGPT’s release. This difference in controversy profile reflects a difference in controversy’s capacity to mobilise AI publics. Deep frictions enable the identification of specific affected communities impacted by particular deployments. The CNAF welfare algorithm in France and the facial recognition deployments in the UK both generated coalitions of identifiable actors (i.e., advocacy organisations, legal challengers, regulatory bodies) that translated controversy into regulatory responses and democratic governance initiatives. Wide frictions, by contrast, distributed concern across many domains simultaneously without catalysing around defined interest groups during this initial period.

The implications of this, however, are differentially distributed across national regulatory architectures. Where horizontal legislation creates formal accountability mechanisms, as the EU AI Act does through its provisions on general-purpose AI, 9 wide controversies create a supportive context for the formalisation of AI governance by governments. The German case illustrates this dynamic at the national level: a robust regulatory architecture channelled governance through institutional structures. However, wide controversies pose special challenges for democratic innovation governance in countries with a laissez-faire approach to AI, as in the United Kingdom (Unver and Odusola-Stevenson 2025), as here civic engagement plays a decisive role in agenda-setting in government policy. Where governance proceeds through sector-specific guidance and pro-innovation frameworks, as in the UK^10^ (Roberts et al. 2023) and Canada (Dandurand et al. 2023), public mobilisation around situated deployments appears to represent a critical governance resource, as the ICO intervention in the UK case illustrated (ICO 2021).

Different waves of AI deployment have thus sparked different forms of AI controversies, and our consultations sit at a hinge moment between them. The late 2010s wave of ML-based systems generated frictions locatable and actionable, tied to named tools, identifiable victims, and institutional actors. By contrast, GAIT and LLMs couplings during our study period made specific points of contention and localised harms much harder to trace. It seems this has begun to change after our consultations had come to an end: more grounded controversies around GAIT have started to take shape, as demonstrated by recent protests by creative industries workers against IP theft by AI companies (Milmo 2026), emerging local mobilisations against data centres construction (Rone 2024), and by exploitative working conditions faced by outsourced data labellers (Klovig Skelton 2025; Perrigo 2023) and high-profile cases, such as GPT-assisted suicide (Yousif 2025). Yet, at the time of the consultations, these harms remained comparatively underpublicised in public discourse and they were not prominently articulated by expert respondents.

## Conclusion

Our consultations surfaced a distinctive moment in the public life of AI, one in which efforts to make AI publicly accountable by focusing on real-world incidents came to be overshadowed by generalist framings of AI following the release of ChatGPT. Such shifts have implications for democratic governance. Situated frictions with deep controversies enable the formation of organised publics around specific deployments; in contrast wide GAIT controversies lacked this situational anchoring of AI controversy in social and public environments in society. Our analysis thus points to the situated controversiality of AI as a generative resource for public involvement in AI controversy: it is a key enabling condition for public problematisation, as the articulation of contexts of application makes it possible for specific uses of AI systems to be examined, evaluated and challenged from society. However, our research also reveals this capacity of AI controversies to generate situated problematisations is fragile and can be easily lost, as was the case in the period immediately following the release of ChatGPT.

Our relational analysis thus tells a “tale of two types of controversy”, in which a plurality of deep contextual problematisations of AI was followed by a large wave of general-purpose technologies, with the wide circulation of more abstract framings across domains. While AI controversies continued to thematise localised contestations of AI during this period, the growing prominence of GAIT asserted a de-contextualising framing of AI, and for a while, it even seemed generative AI would come to stand in for AI as a whole, failing to catalyse the kinds of engaged processes of public problem articulation seen with earlier waves, at least initially. Whether this represents a lasting transformation in the structure of AI controversiality or a historically bounded pattern of submersion, in which a larger wave temporarily drowns out the frictions accumulated during an earlier cycle before these resurface in new form, is a question this study cannot resolve, but which it makes visible.

Being a study at the threshold of a transformation in AI adoption in society, it captures a field of controversy in the moment of its recomposition. This also generates methodological tensions in our approach. First, our standpoint approach foregrounds contextual specificity and situated expertise, yet our central finding operates at a level of analytical generality that might seem to contradict this commitment. We affirm this as a productive methodological tension rather than a limitation to be resolved. The general shift we detected presents an *emergent, relational pattern* observed across multiple standpoints. We identified these logics through comparative analysis across contexts, grounded in the specific expert articulations within each national consultation. It also shows our emphasis on linking AI controversies to particular places, events and contexts does not limit analytical scope. Instead, it highlights the distributed character of AI controversiality, emerging from multiple situated sites simultaneously, rather than diffusing from a single abstract framing as occurs with wide GAIT controversies. This distributed approach to controversy analysis affirms there are multiple entry points for contestation tied to lived experience, and, as such, enabled us to evaluate conditions for democratic governance of AI (Chilvers and Kearnes 2020).

Second, the events at which we recruited our extended expert communities shape which expert voices are represented in each consultation, and some of the cross-national differences may reflect variation in event types, but this makes the patterns we identified no less analytically significant. Rather, they reveal *how issue articulation operates within differently configured epistemic communities*. Different types of events attract different expert communities, and these communities develop distinct registers for articulating AI controversiality. Our findings thus can help shed light on *divergent logics of problematisation* across national contexts, a substantive finding about how AI governance is actually enacted.

The public sites of AI expert engagement that we selected were, at the time of this study, among the primary sites where AI was being publicly constituted as a societal concern across the four national contexts. These sites were not exhaustive of all AI-related expertise and notably did not yet capture the broader practitioner communities that have since emerged. However, this strategy was informed by the relative absence of other organised communities through which situated engagements with AI could be rendered legible as AI & Society controversies. More generally, after ChatGPT, the transition from deep to wide controversy also marked an expansion of the controversy landscape itself: more communities became implicated, but this broader involvement did not necessarily produce deeper problematisation.

Future research could evaluate the possibility of a new wave in AI controversiality, as in the years following our consultation, affected communities organised to challenge the social, cultural and economic impacts of GAIT, in the creative industries, higher education and the health sector. This would enable a new assessment of the inclusivity of the epistemic communities mobilised around GAITs. Equally important would be to consider whether and how GAIT technologies themselves have become ‘grounded’ through incidents in specific contexts over time. Such research would help clarify whether the deep-to-wide shift we observed represents a lasting recomposition of AI controversiality, or a transitional phase as new technologies mature and their societal implications become more tangible and contestable. We expect, however, that democratic AI governance will continue to depend on the situational articulation of AI frictions, as key to the demonstration of AI’s varied impacts across society and to enable the mobilisation of affected publics.

## Appendix

See Tables 4, 5

**Table 4:** Friction object categories with illustrative examples cited across consultation data. Examples represent key technologies or cases associated with each group

Frictions grouped	Examples of frictions
AI Assistant (Siri, Alexa)	Amazon Echo, Google Assistant (haircut appointment demo), BlenderBot, virtual assistants, Siri, Alexa, Roomba, Sophia (humanoid robot granted citizenship)
Alphago	AlphaGo vs. Lee Sedol, Deep Blue defeats Kasparov, IBM Watson wins Jeopardy
Autonomous cars, drones & robots (Testla, Maven)	Tesla crashes, Boston Dynamics robots, semi-autonomous cars on public roads, DARPA projects, Project Maven, Slaughterbots video, military drones, autonomous weapons systems
Charismatic opinions (Open Letter, Hinton Resignment)	Geoffrey Hinton’s resignation, Open Letter calling for a 6-month pause on AI development, Hawking’s warning, Yuval Noah Harari’s superintelligence arguments, sci-fi references
Corporate power & culture (Gebru, parrot paper)	Google and OpenAI dominance, Amazon worker strikes, Timnit Gebru and Margaret Mitchell firings, Stochastic Parrots paper
Decision-making tools (Syri, amazon hiring tool)	Amazon hiring algorithm (gender bias), CV evaluation tools, UK exam grading algorithm (“Exams fiasco”), Syri (Netherlands)
Financial & business systems (Vital, insurances)	AI in finance, predictive insurance models, health insurance, Vital financial tools
FRT intersectional & systemic errors (Gaydar, Amazon Rekognition)	Amazon Rekognition, Gender Shades study, Gaydar AI, Nikon facial detection issues, Gorilla image-tagging incident, Flickr dataset scraping (IBM)
Healthcare & diagnostics (Oncofinder, Digital Twin)	Google’s Project Nightingale, DeepMind-NHS partnership, NHS Care.data project, OncoFinder, Sloan Kettering Cancer Center diagnostics, digital twins in health
Image Generation Tools (Dall E, MIDJOURNEY)	DALL·E, Midjourney (Colorado art contest), Stable Diffusion, CLIP, Codex
Internal & External Social Sorting (social credit Score, immigration control)	Chinese social credit system, iBorderControl, biometric border checks, UK Home Office passport AI, predictive risk assessments for “at-risk” families
LLM Products & Companies (Openai, GPT)	ChatGPT, GPT-3, GPT (various versions), OpenAI, GenAI in school, click workers
Media content generation & diffusion (deep fakes, recommendation algorithms)	StyleGAN, Neuralink, Deepfakes (Putin, Obama, porn), Facebook/YouTube/TikTok algorithms, fake news, emotional AI, The Cleaners (content moderation)
Political Manipulation (Cambridge Analytica, Mark Zuckerberg’s Hearing)	Cambridge Analytica, Brexit, Zuckerberg Senate hearing, bots and disinformation, #MeToo, U.S. Capitol riot, Musk’s Twitter takeover
Regulation (Ai Act, Rgpd)	EU AI Act, GDPR, German AI standardisation roadmap, German Data Ethics Commission, UK AI Strategy, AI Enquete Commission (Germany),
STS studies (Cloud Ethics, Zylinska)	Zylinska’s aesthetics, Amoore’s *Cloud Ethics*, fairness discourse, ADM system critiques, energy impacts of NLP, DFKI. ISO standards
Surveillance & monitoring systems (Clearview AI, Uighurs, King’s Cross)	Clearview AI, facial recognition at King’s Cross (London), FRT for lunch payments in schools in Ayrshire (Scotland), COVID tracking in China (SenseTime), Chinese police sunglasses, predictive policing, Palantir and LAPD, R (Bridges) v Chief Constable of South Wales (UK legal case), Marseille high school FRT use, urban surveillance systems, FRT in German train stations
US Law & court AI tools (Compas)	COMPAS risk assessment tool, algorithmic decision-making in US judicial system, law and jurisprudence applications

**Table 5:** Topics categories with illustrative examples cited across consultation data. Examples represent key technologies or cases associated with each group

Topics grouped	Examples of topics
AI & democracy (Mis, Disinformation)	Disinformation, public communications, social risk, trust, democratic processes
Automation (Human agency, HM interaction)	Autonomous machines, human–machine interaction
Computational infrastructures (Data, Scale)	Data infrastructures, scale, accountability, bias, power
Creativity & intellectual property (Copyright,?)	Creativity and artistic content, copyright, authorship, intellectual property
Ethical & social justice issues (Bias, Ethics)	Bias, discrimination, accountability, privacy, justice, social harm, surveillance
Future risks & social harms (Existential threats, AGI)	AGI, existential risk, long-term harm, social risk, trust, state power
Governance & trust (regulation, oversight)	Regulation, oversight, power, surveillance, standard-setting, privacy
Impacts of AI (work, environment)	Labour, environment, war, health
ML epistemology (AI definition, learning)	ML Epistemology, explainability, knowledge, definitions, bias
State & corporate power Dynamics	Power, public–private partnerships, governmentality and governance, surveillance, immigration, regulation
Surveillance & security (Law enforcement, Profiling)	Predictive policing, profiling, human tracking, security tech
AI & social life	Everyday life, society, social norms, public interactions

## Data Availability

Data generated and analysed during this study are subject to varying data governance and ethical requirements across the participating countries. As a result, the datasets are not publicly available. However, data access may be granted on reasonable request, subject to approval by the relevant research teams and in accordance with their institutional ethics protocols. All research activities conducted as part of this study received ethical approval from the relevant Ethics Committees or Institutional Review Boards at the respective institutions of the participating research teams. Each team was responsible for securing approval in accordance with their national and institutional requirements for research involving human participants. Informed consent to participate was obtained by each research team in line with their approved ethical protocols.

## Footnotes

1 Policy makers, politicians, civil servants, experts and journalists increasingly turned to AI to create, analyse and report on public policy. AI tools are increasingly deployed in journalism, raising concerns about editorial accountability, bias, and misinformation and journalism’s democratic role as a space for critical debate (UNRIC 2025). Meanwhile, GAIT has entered the policy process itself. In the UK, the Technology Secretary reportedly used ChatGPT to draft speeches (Stokel-Walker 2025), and the UK Prime Minister has proposed replacing some civil service tasks with generative AI, further embedding GAIT in institutional workflows (Mason 2025).

2 The second question was designed as a prompt to provide starting points for media analysis, enabling us to extend our controversy elicitation into online environments.

3 This article focuses on the socio-centric consultation to foreground societal perspectives and public framings of AI controversies. A comparative analysis involving the science-centric consultation, which examines internal perspectives from within the AI research community, will be presented in a separate publication.

4 For the UK: Alan Turing Institute UK AI Showcase (2021), Ada Lovelace at CogX (2021), Oxford AI Ethics Futures (2021), Ada Lovelace Institute Data Stewardship (2021), Mozilla Fest (2021); ArtAI Festival (2021); AI Summit (2021); UAL Tech and Power (2021), AI World Congress (2021). For France Big Data & AI Paris (2022), PFIA—Plate-forme Intelligence Artificielle (2021, 2022), AI France Summit (2022). For Canada: Big Data & AI Toronto (2020–2023), Montreal AI Symposium (multiple years), Vector Institute AI Summit & Career Fair (2021–2022), Women in AI Summit (2020), University of Toronto AI Conference (2022–2023), York University’s Bracing for Impact series (2020–2023), Fields Institute Machine Learning Advances Seminar (2019–2023), and programming by leading institutions such as MILA, AMII, IVADO, and OBVIA and major university AI hubs (e.g., University of Toronto, University of British Columbia, Simon Fraser University, University of Alberta). For Germany: Federal Ministry of Labour’s AICamp 2021, Responsible AI Forum (2021–22), Annual Conferences of the High-Level Network “Plattform Lernende Systeme” (2021–22), Digital Society Festival re:publica (2021–22), University of Bonn’s Sustainable AI Conference (2021).

5 For comprehensive lists of all friction and topic categories with detailed examples, see supplementary tables 4 and 5 in the appendix.

6 A topic and a friction were coded as co-occurring when they were mentioned by one and the same respondent and deemed thematically related by the human coder of the responses.

7 This technique, adapted from information retrieval and text mining helps distinguish locally prominent associations from globally generic ones. Term Frequency (TF) measures how central a specific coupling is to a country’s consultation responses, while Inverse Document Frequency (IDF) downweighs pairs that occur commonly across countries. The resulting TF-IDF score thus identifies which couplings are not only frequent but also distinctive, surfacing context-sensitive issue configurations that may otherwise be obscured by globally dominant narratives.

8 We assume that this finding is indicative of both a country-specific framing of AI controversiality in society-centred expert communities as well as of the profiles of respondents. We have followed the same procedure to identify experts as in other countries to avoid sampling being defined by personal networks of researchers. But the response might have been higher with experts engaged in the long-standing extended expert community with the society-centred aspect of the digital transformation for over a decade and that consists of both academics and NGOs.

9 For examples of regulatory responses to GAIT: the EU’s AI Act includes specific provisions on foundation models and GPAI; France’s CNIL issued guidance on generative models and data protection (CNIL 2024).

10 In the UK, a pro-innovation approach has so far avoided horizontal legislation, with guidance emerging instead from bodies like the Information Commissioner’s Office (ICO 2024).
